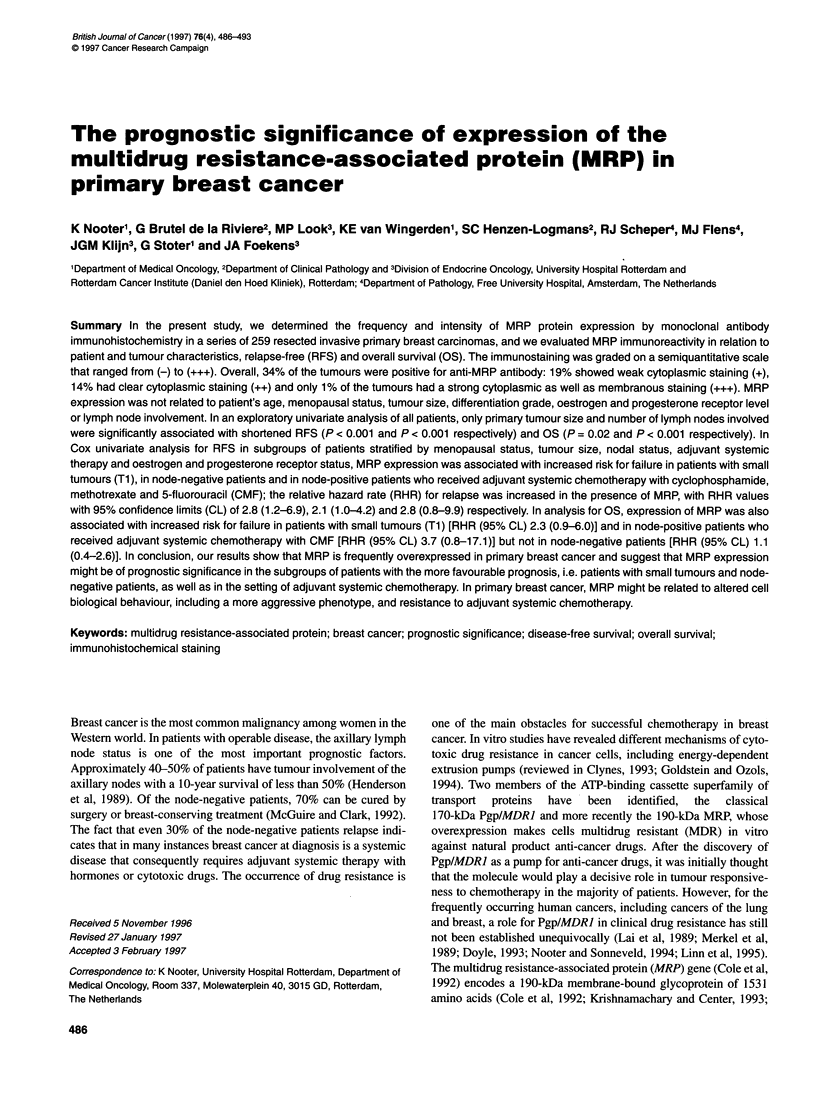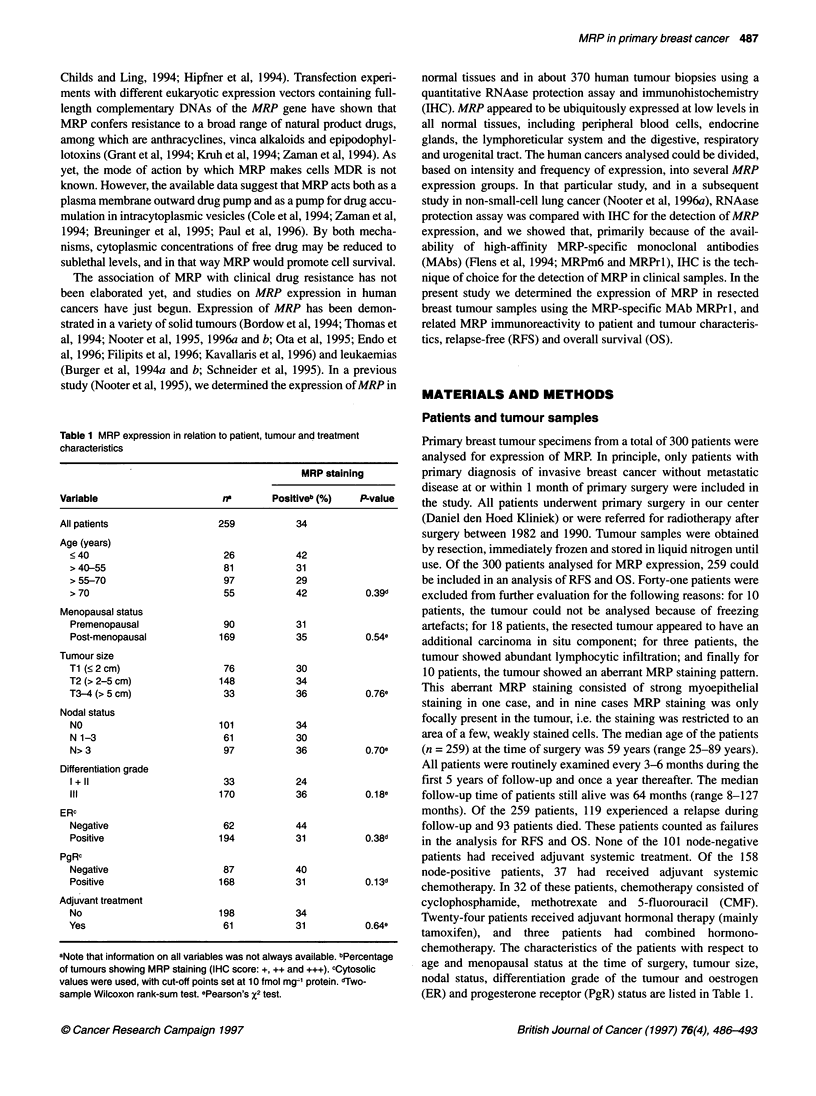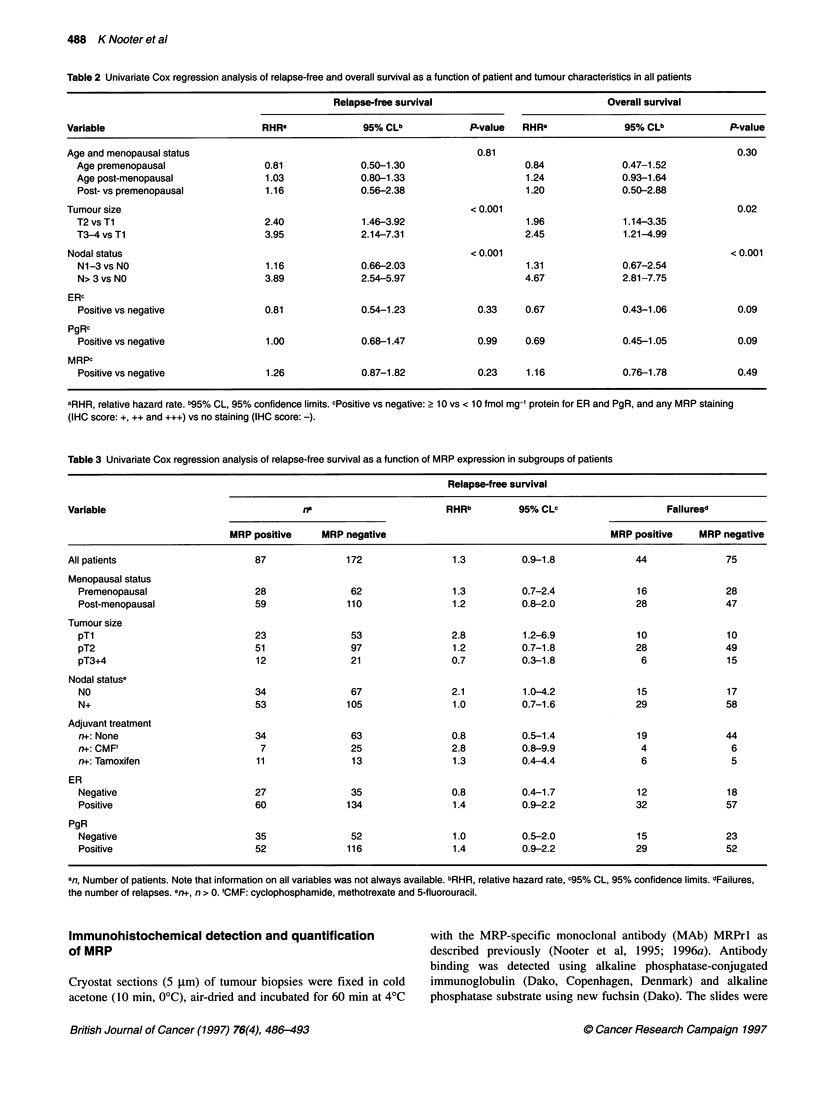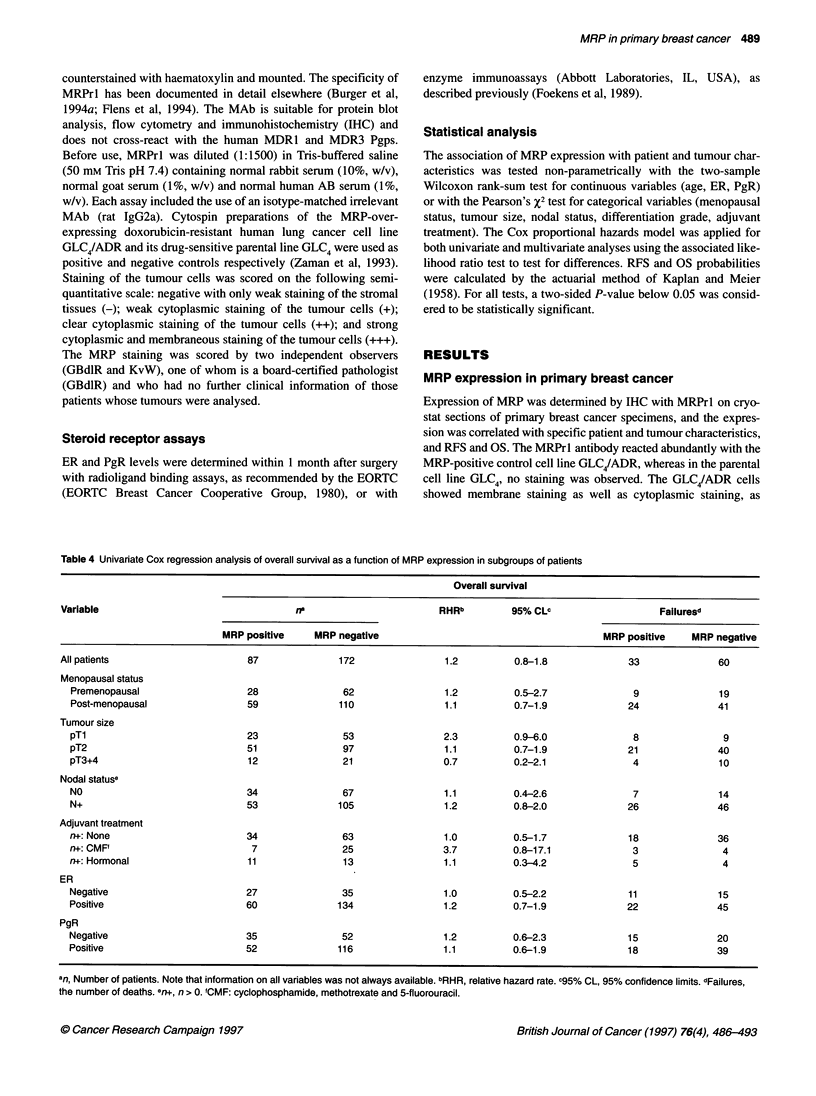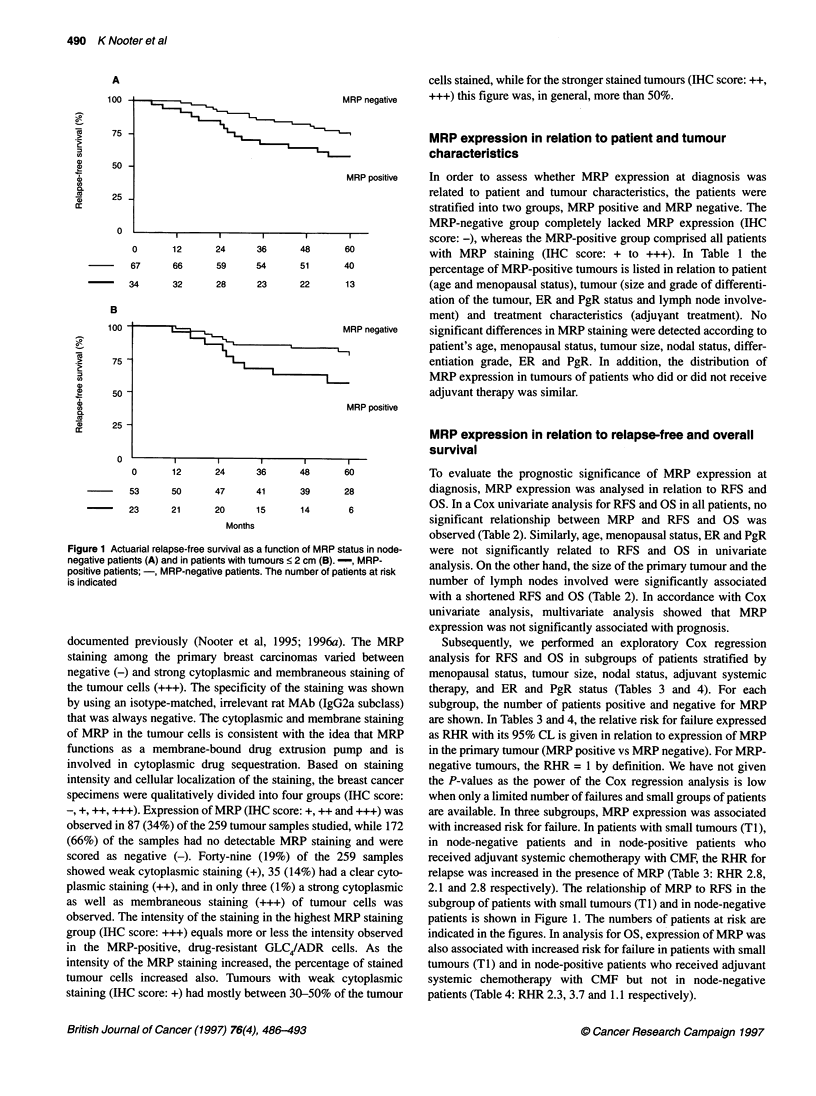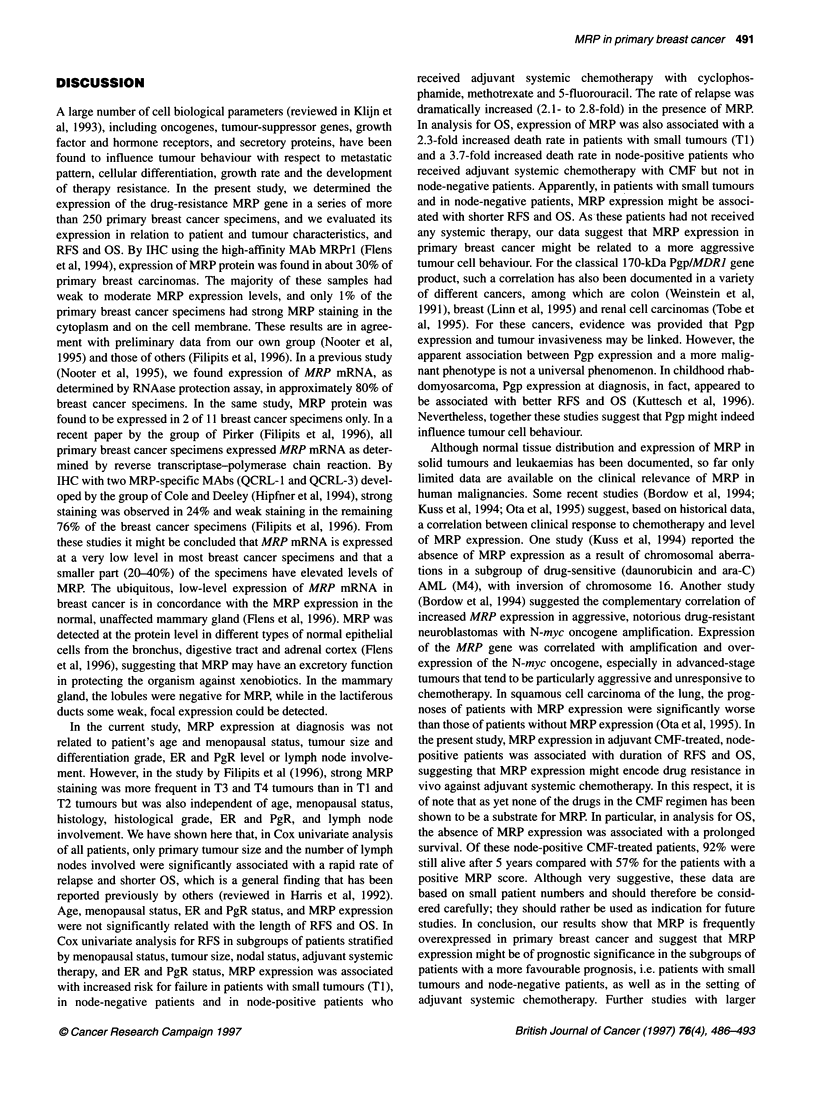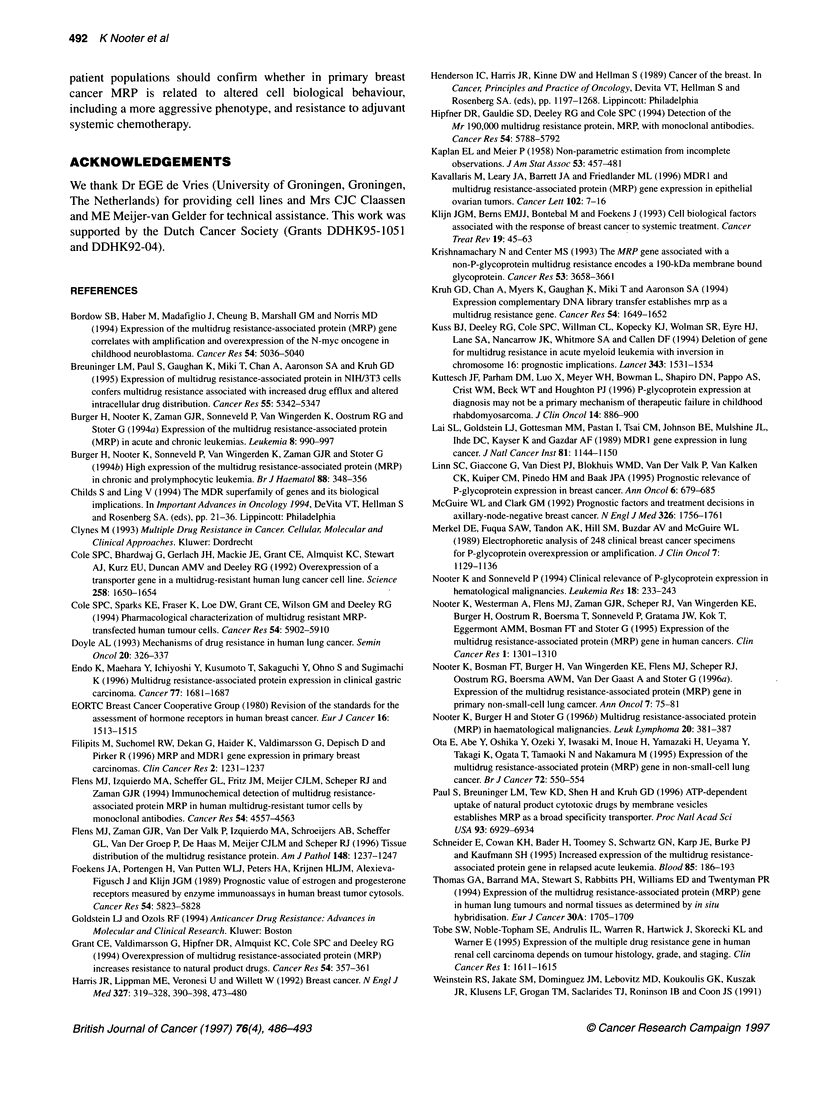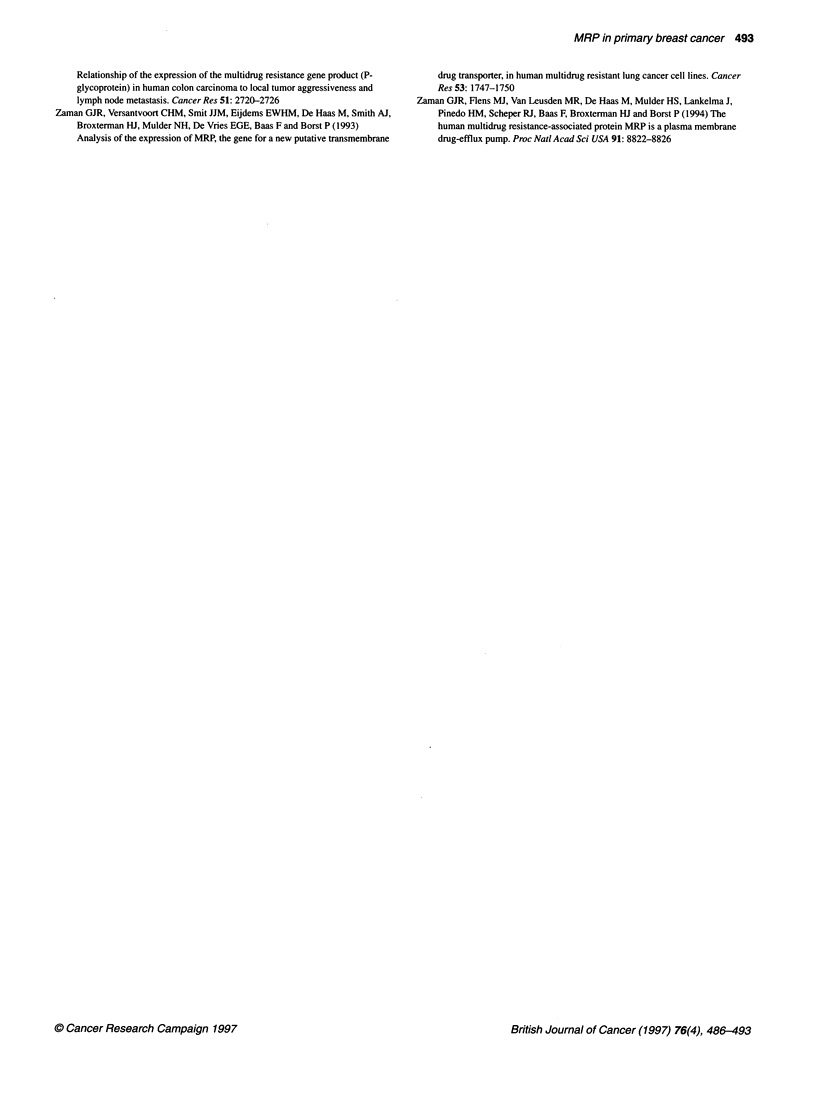# The prognostic significance of expression of the multidrug resistance-associated protein (MRP) in primary breast cancer.

**DOI:** 10.1038/bjc.1997.414

**Published:** 1997

**Authors:** K. Nooter, G. Brutel de la Riviere, M. P. Look, K. E. van Wingerden, S. C. Henzen-Logmans, R. J. Scheper, M. J. Flens, J. G. Klijn, G. Stoter, J. A. Foekens

**Affiliations:** Department of Medical Oncology, University Hospital Rotterdam and Rotterdam Cancer Institute (Daniel den Hoed Kliniek), The Netherlands.

## Abstract

In the present study, we determined the frequency and intensity of MRP protein expression by monoclonal antibody immunohistochemistry in a series of 259 resected invasive primary breast carcinomas, and we evaluated MRP immunoreactivity in relation to patient and tumour characteristics, relapse-free (RFS) and overall survival (OS). The immunostaining was graded on a semiquantitative scale that ranged from (-) to ( ). Overall, 34% of the tumours were positive for anti-MRP antibody: 19% showed weak cytoplasmic staining (+), 14% had clear cytoplasmic staining (++) and only 1% of the tumours had a strong cytoplasmic as well as membranous staining ( ). MRP expression was not related to patient's age, menopausal status, tumour size, differentiation grade, oestrogen and progesterone receptor level or lymph node involvement. In an exploratory univariate analysis of all patients, only primary tumour size and number of lymph nodes involved were significantly associated with shortened RFS (P < 0.001 and P < 0.001 respectively) and OS (P = 0.02 and P < 0.001 respectively). In Cox univariate analysis for RFS in subgroups of patients stratified by menopausal status, tumour size, nodal status, adjuvant systemic therapy and oestrogen and progesterone receptor status, MRP expression was associated with increased risk for failure in patients with small tumours (T1), in node-negative patients and in node-positive patients who received adjuvant systemic chemotherapy with cyclophosphamide, methotrexate and 5-fluorouracil (CMF); the relative hazard rate (RHR) for relapse was increased in the presence of MRP, with RHR values with 95% confidence limits (CL) of 2.8 (1.2-6.9), 2.1 (1.0-4.2) and 2.8 (0.8-9.9) respectively. In analysis for OS, expression of MRP was also associated with increased risk for failure in patients with small tumours (T1) [RHR (95% CL) 2.3 (0.9-6.0)] and in node-positive patients who received adjuvant systemic chemotherapy with CMF [RHR (95% CL) 3.7 (0.8-17.1)] but not in node-negative patients [RHR (95% CL) 1.1 (0.4-2.6)]. In conclusion, our results show that MRP is frequently overexpressed in primary breast cancer and suggest that MRP expression might be of prognostic significance in the subgroups of patients with the more favourable prognosis, i.e. patients with small tumours and node-negative patients, as well as in the setting of adjuvant systemic chemotherapy. In primary breast cancer, MRP might be related to altered cell biological behaviour, including a more aggressive phenotype, and resistance to adjuvant systemic chemotherapy.


*British Journal of Cancer* (1997) **76**(4), 486–493
© 1997 Cancer Research Campaign

### The prognostic significance of expression of the multidrug resistance-associated protein (MRP) in primary breast cancer

**K Nooter[1], G Brutel de la Riviere[2], MP Look[3], KE van Wingerden[1], SC Henzen-Logmans[2], RJ Scheper[4], MJ Flens[4], JGM Klijn[3], G Stoter[1] and JA Foekens[3]**

[1]Department of Medical Oncology, [2]Department of Clinical Pathology and [3]Division of Endocrine Oncology, University Hospital Rotterdam and Rotterdam Cancer Institute (Daniel den Hoed Kliniek), Rotterdam; [4]Department of Pathology, Free University Hospital, Amsterdam, The Netherlands

**Summary** In the present study, we determined the frequency and intensity of MRP protein expression by monoclonal antibody immunohistochemistry in a series of 259 resected invasive primary breast carcinomas, and we evaluated MRP immunoreactivity in relation to patient and tumour characteristics, relapse-free (RFS) and overall survival (OS). The immunostaining was graded on a semiquantitative scale that ranged from (–) to (+++). Overall, 34% of the tumours were positive for anti-MRP antibody: 19% showed weak cytoplasmic staining (+), 14% had clear cytoplasmic staining (++) and only 1% of the tumours had a strong cytoplasmic as well as membranous staining (+++). MRP expression was not related to patient's age, menopausal status, tumour size, differentiation grade, oestrogen and progesterone receptor level or lymph node involvement. In an exploratory univariate analysis of all patients, only primary tumour size and number of lymph nodes involved were significantly associated with shortened RFS ($P < 0.001$ and $P < 0.001$ respectively) and OS ($P = 0.02$ and $P < 0.001$ respectively). In Cox univariate analysis for RFS in subgroups of patients stratified by menopausal status, tumour size, nodal status, adjuvant systemic therapy and oestrogen and progesterone receptor status, MRP expression was associated with increased risk for failure in patients with small tumours (T1), in node-negative patients and in node-positive patients who received adjuvant systemic chemotherapy with cyclophosphamide, methotrexate and 5-fluorouracil (CMF); the relative hazard rate (RHR) for relapse was increased in the presence of MRP, with RHR values with 95% confidence limits (CL) of 2.8 (1.2–6.9), 2.1 (1.0–4.2) and 2.8 (0.8–9.9) respectively. In analysis for OS, expression of MRP was also associated with increased risk for failure in patients with small tumours (T1) [RHR (95% CL) 2.3 (0.9–6.0)] and in node-positive patients who received adjuvant systemic chemotherapy with CMF [RHR (95% CL) 3.7 (0.8–17.1)] but not in node-negative patients [RHR (95% CL) 1.1 (0.4–2.6)]. In conclusion, our results show that MRP is frequently overexpressed in primary breast cancer and suggest that MRP expression might be of prognostic significance in the subgroups of patients with the more favourable prognosis, i.e. patients with small tumours and node-negative patients, as well as in the setting of adjuvant systemic chemotherapy. In primary breast cancer, MRP might be related to altered cell biological behaviour, including a more aggressive phenotype, and resistance to adjuvant systemic chemotherapy.

**Keywords:** multidrug resistance-associated protein; breast cancer; prognostic significance; disease-free survival; overall survival; immunohistochemical staining

Breast cancer is the most common malignancy among women in the Western world. In patients with operable disease, the axillary lymph node status is one of the most important prognostic factors. Approximately 40–50% of patients have tumour involvement of the axillary nodes with a 10-year survival of less than 50% (Henderson et al, 1989). Of the node-negative patients, 70% can be cured by surgery or breast-conserving treatment (McGuire and Clark, 1992). The fact that even 30% of the node-negative patients relapse indicates that in many instances breast cancer at diagnosis is a systemic disease that consequently requires adjuvant systemic therapy with hormones or cytotoxic drugs. The occurrence of drug resistance is one of the main obstacles for successful chemotherapy in breast cancer. In vitro studies have revealed different mechanisms of cytotoxic drug resistance in cancer cells, including energy-dependent extrusion pumps (reviewed in Clynes, 1993; Goldstein and Ozols, 1994). Two members of the ATP-binding cassette superfamily of transport proteins have been identified, the classical 170-kDa Pgp/*MDR1* and more recently the 190-kDa MRP, whose overexpression makes cells multidrug resistant (MDR) in vitro against natural product anti-cancer drugs. After the discovery of Pgp/*MDR1* as a pump for anti-cancer drugs, it was initially thought that the molecule would play a decisive role in tumour responsiveness to chemotherapy in the majority of patients. However, for the frequently occurring human cancers, including cancers of the lung and breast, a role for Pgp/*MDR1* in clinical drug resistance has still not been established unequivocally (Lai et al, 1989; Merkel et al, 1989; Doyle, 1993; Nooter and Sonneveld, 1994; Linn et al, 1995). The multidrug resistance-associated protein (*MRP*) gene (Cole et al, 1992) encodes a 190-kDa membrane-bound glycoprotein of 1531 amino acids (Cole et al, 1992; Krishnamachary and Center, 1993;

*Received 5 November 1996*
*Revised 27 January 1997*
*Accepted 3 February 1997*

*Correspondence to:* K Nooter, University Hospital Rotterdam, Department of Medical Oncology, Room 337, Molewaterplein 40, 3015 GD, Rotterdam, The Netherlands

Childs and Ling, 1994; Hipfner et al, 1994). Transfection experiments with different eukaryotic expression vectors containing full-length complementary DNAs of the *MRP* gene have shown that MRP confers resistance to a broad range of natural product drugs, among which are anthracyclines, vinca alkaloids and epipodophyllotoxins (Grant et al, 1994; Kruh et al, 1994; Zaman et al, 1994). As yet, the mode of action by which MRP makes cells MDR is not known. However, the available data suggest that MRP acts both as a plasma membrane outward drug pump and as a pump for drug accumulation in intracytoplasmic vesicles (Cole et al, 1994; Zaman et al, 1994; Breuninger et al, 1995; Paul et al, 1996). By both mechanisms, cytoplasmic concentrations of free drug may be reduced to sublethal levels, and in that way MRP would promote cell survival.

The association of MRP with clinical drug resistance has not been elaborated yet, and studies on *MRP* expression in human cancers have just begun. Expression of *MRP* has been demonstrated in a variety of solid tumours (Bordow et al, 1994; Thomas et al, 1994; Nooter et al, 1995, 1996*a* and *b*; Ota et al, 1995; Endo et al, 1996; Filipits et al, 1996; Kavallaris et al, 1996) and leukaemias (Burger et al, 1994*a* and *b*; Schneider et al, 1995). In a previous study (Nooter et al, 1995), we determined the expression of *MRP* in normal tissues and in about 370 human tumour biopsies using a quantitative RNAase protection assay and immunohistochemistry (IHC). *MRP* appeared to be ubiquitously expressed at low levels in all normal tissues, including peripheral blood cells, endocrine glands, the lymphoreticular system and the digestive, respiratory and urogenital tract. The human cancers analysed could be divided, based on intensity and frequency of expression, into several *MRP* expression groups. In that particular study, and in a subsequent study in non-small-cell lung cancer (Nooter et al, 1996*a*), RNAase protection assay was compared with IHC for the detection of *MRP* expression, and we showed that, primarily because of the availability of high-affinity MRP-specific monoclonal antibodies (MAbs) (Flens et al, 1994; MRPm6 and MRPr1), IHC is the technique of choice for the detection of MRP in clinical samples. In the present study we determined the expression of MRP in resected breast tumour samples using the MRP-specific MAb MRPr1, and related MRP immunoreactivity to patient and tumour characteristics, relapse-free (RFS) and overall survival (OS).

**Table 1** MRP expression in relation to patient, tumour and treatment characteristics

| Variable | n[a] | MRP staining: Positive[b] (%) | MRP staining: P-value |
|---|---|---|---|
| All patients | 259 | 34 | |
| Age (years) | | | |
| ≤ 40 | 26 | 42 | |
| > 40–55 | 81 | 31 | |
| > 55–70 | 97 | 29 | |
| > 70 | 55 | 42 | 0.39[d] |
| Menopausal status | | | |
| Premenopausal | 90 | 31 | |
| Post-menopausal | 169 | 35 | 0.54[e] |
| Tumour size | | | |
| T1 (≤ 2 cm) | 76 | 30 | |
| T2 (> 2–5 cm) | 148 | 34 | |
| T3–4 (> 5 cm) | 33 | 36 | 0.76[e] |
| Nodal status | | | |
| N0 | 101 | 34 | |
| N 1–3 | 61 | 30 | |
| N> 3 | 97 | 36 | 0.70[e] |
| Differentiation grade | | | |
| I + II | 33 | 24 | |
| III | 170 | 36 | 0.18[e] |
| ER[c] | | | |
| Negative | 62 | 44 | |
| Positive | 194 | 31 | 0.38[d] |
| PgR[c] | | | |
| Negative | 87 | 40 | |
| Positive | 168 | 31 | 0.13[d] |
| Adjuvant treatment | | | |
| No | 198 | 34 | |
| Yes | 61 | 31 | 0.64[e] |

[a]Note that information on all variables was not always available. [b]Percentage of tumours showing MRP staining (IHC score: +, ++ and +++). [c]Cytosolic values were used, with cut-off points set at 10 fmol $mg^{-1}$ protein. [d]Two-sample Wilcoxon rank-sum test. [e]Pearson's $\chi^2$ test.

### MATERIALS AND METHODS

### Patients and tumour samples

Primary breast tumour specimens from a total of 300 patients were analysed for expression of MRP. In principle, only patients with primary diagnosis of invasive breast cancer without metastatic disease at or within 1 month of primary surgery were included in the study. All patients underwent primary surgery in our center (Daniel den Hoed Kliniek) or were referred for radiotherapy after surgery between 1982 and 1990. Tumour samples were obtained by resection, immediately frozen and stored in liquid nitrogen until use. Of the 300 patients analysed for MRP expression, 259 could be included in an analysis of RFS and OS. Forty-one patients were excluded from further evaluation for the following reasons: for 10 patients, the tumour could not be analysed because of freezing artefacts; for 18 patients, the resected tumour appeared to have an additional carcinoma in situ component; for three patients, the tumour showed abundant lymphocytic infiltration; and finally for 10 patients, the tumour showed an aberrant MRP staining pattern. This aberrant MRP staining consisted of strong myoepithelial staining in one case, and in nine cases MRP staining was only focally present in the tumour, i.e. the staining was restricted to an area of a few, weakly stained cells. The median age of the patients ($n$ = 259) at the time of surgery was 59 years (range 25–89 years). All patients were routinely examined every 3–6 months during the first 5 years of follow-up and once a year thereafter. The median follow-up time of patients still alive was 64 months (range 8–127 months). Of the 259 patients, 119 experienced a relapse during follow-up and 93 patients died. These patients counted as failures in the analysis for RFS and OS. None of the 101 node-negative patients had received adjuvant systemic treatment. Of the 158 node-positive patients, 37 had received adjuvant systemic chemotherapy. In 32 of these patients, chemotherapy consisted of cyclophosphamide, methotrexate and 5-fluorouracil (CMF). Twenty-four patients received adjuvant hormonal therapy (mainly tamoxifen), and three patients had combined hormono-chemotherapy. The characteristics of the patients with respect to age and menopausal status at the time of surgery, tumour size, nodal status, differentiation grade of the tumour and oestrogen (ER) and progesterone receptor (PgR) status are listed in Table 1.

© Cancer Research Campaign 1997 

Table 2 Univariate Cox regression analysis of relapse-free and overall survival as a function of patient and tumour characteristics in all patients

| Variable | Relapse-free survival RHR[a] | Relapse-free survival 95% CL[b] | Relapse-free survival P-value | Overall survival RHR[a] | Overall survival 95% CL[b] | Overall survival P-value |
|---|---|---|---|---|---|---|
| Age and menopausal status | | | 0.81 | | | 0.30 |
| Age premenopausal | 0.81 | 0.50–1.30 | | 0.84 | 0.47–1.52 | |
| Age post-menopausal | 1.03 | 0.80–1.33 | | 1.24 | 0.93–1.64 | |
| Post- vs premenopausal | 1.16 | 0.56–2.38 | | 1.20 | 0.50–2.88 | |
| Tumour size | | | < 0.001 | | | 0.02 |
| T2 vs T1 | 2.40 | 1.46–3.92 | | 1.96 | 1.14–3.35 | |
| T3–4 vs T1 | 3.95 | 2.14–7.31 | | 2.45 | 1.21–4.99 | |
| Nodal status | | | < 0.001 | | | < 0.001 |
| N1–3 vs N0 | 1.16 | 0.66–2.03 | | 1.31 | 0.67–2.54 | |
| N> 3 vs N0 | 3.89 | 2.54–5.97 | | 4.67 | 2.81–7.75 | |
| ER[c] | | | | | | |
| Positive vs negative | 0.81 | 0.54–1.23 | 0.33 | 0.67 | 0.43–1.06 | 0.09 |
| PgR[c] | | | | | | |
| Positive vs negative | 1.00 | 0.68–1.47 | 0.99 | 0.69 | 0.45–1.05 | 0.09 |
| MRP[c] | | | | | | |
| Positive vs negative | 1.26 | 0.87–1.82 | 0.23 | 1.16 | 0.76–1.78 | 0.49 |

[a]RHR, relative hazard rate. [b]95% CL, 95% confidence limits. [c]Positive vs negative: ≥ 10 vs < 10 fmol mg$^{-1}$ protein for ER and PgR, and any MRP staining (IHC score: +, ++ and +++) vs no staining (IHC score: –).

Table 3 Univariate Cox regression analysis of relapse-free survival as a function of MRP expression in subgroups of patients

| Variable | n[a] MRP positive | n[a] MRP negative | RHR[b] | 95% CL[c] | Failures[d] MRP positive | Failures[d] MRP negative |
|---|---|---|---|---|---|---|
| All patients | 87 | 172 | 1.3 | 0.9–1.8 | 44 | 75 |
| Menopausal status | | | | | | |
| Premenopausal | 28 | 62 | 1.3 | 0.7–2.4 | 16 | 28 |
| Post-menopausal | 59 | 110 | 1.2 | 0.8–2.0 | 28 | 47 |
| Tumour size | | | | | | |
| pT1 | 23 | 53 | 2.8 | 1.2–6.9 | 10 | 10 |
| pT2 | 51 | 97 | 1.2 | 0.7–1.8 | 28 | 49 |
| pT3+4 | 12 | 21 | 0.7 | 0.3–1.8 | 6 | 15 |
| Nodal status[e] | | | | | | |
| N0 | 34 | 67 | 2.1 | 1.0–4.2 | 15 | 17 |
| N+ | 53 | 105 | 1.0 | 0.7–1.6 | 29 | 58 |
| Adjuvant treatment | | | | | | |
| n+: None | 34 | 63 | 0.8 | 0.5–1.4 | 19 | 44 |
| n+: CMF[f] | 7 | 25 | 2.8 | 0.8–9.9 | 4 | 6 |
| n+: Tamoxifen | 11 | 13 | 1.3 | 0.4–4.4 | 6 | 5 |
| ER | | | | | | |
| Negative | 27 | 35 | 0.8 | 0.4–1.7 | 12 | 18 |
| Positive | 60 | 134 | 1.4 | 0.9–2.2 | 32 | 57 |
| PgR | | | | | | |
| Negative | 35 | 52 | 1.0 | 0.5–2.0 | 15 | 23 |
| Positive | 52 | 116 | 1.4 | 0.9–2.2 | 29 | 52 |

[a]n, Number of patients. Note that information on all variables was not always available. [b]RHR, relative hazard rate, [c]95% CL, 95% confidence limits. [d]Failures, the number of relapses. [e]n+, n > 0. [f]CMF: cyclophosphamide, methotrexate and 5-fluorouracil.

### Immunohistochemical detection and quantification of MRP

Cryostat sections (5 μm) of tumour biopsies were fixed in cold acetone (10 min, 0°C), air-dried and incubated for 60 min at 4°C with the MRP-specific monoclonal antibody (MAb) MRPr1 as described previously (Nooter et al, 1995; 1996*a*). Antibody binding was detected using alkaline phosphatase-conjugated immunoglobulin (Dako, Copenhagen, Denmark) and alkaline phosphatase substrate using new fuchsin (Dako). The slides were

 © Cancer Research Campaign 1997

counterstained with haematoxylin and mounted. The specificity of MRPr1 has been documented in detail elsewhere (Burger et al, 1994*a*; Flens et al, 1994). The MAb is suitable for protein blot analysis, flow cytometry and immunohistochemistry (IHC) and does not cross-react with the human MDR1 and MDR3 Pgps. Before use, MRPr1 was diluted (1:1500) in Tris-buffered saline (50 mM Tris pH 7.4) containing normal rabbit serum (10%, w/v), normal goat serum (1%, w/v) and normal human AB serum (1%, w/v). Each assay included the use of an isotype-matched irrelevant MAb (rat IgG2a). Cytospin preparations of the MRP-over-expressing doxorubicin-resistant human lung cancer cell line $GLC_4$/ADR and its drug-sensitive parental line $GLC_4$ were used as positive and negative controls respectively (Zaman et al, 1993). Staining of the tumour cells was scored on the following semi-quantitative scale: negative with only weak staining of the stromal tissues (–); weak cytoplasmic staining of the tumour cells (+); clear cytoplasmic staining of the tumour cells (++); and strong cytoplasmic and membraneous staining of the tumour cells (+++). The MRP staining was scored by two independent observers (GBdlR and KvW), one of whom is a board-certified pathologist (GBdlR) and who had no further clinical information of those patients whose tumours were analysed.

### Steroid receptor assays

ER and PgR levels were determined within 1 month after surgery with radioligand binding assays, as recommended by the EORTC (EORTC Breast Cancer Cooperative Group, 1980), or with enzyme immunoassays (Abbott Laboratories, IL, USA), as described previously (Foekens et al, 1989).

### Statistical analysis

The association of MRP expression with patient and tumour characteristics was tested non-parametrically with the two-sample Wilcoxon rank-sum test for continuous variables (age, ER, PgR) or with the Pearson's $\chi^2$ test for categorical variables (menopausal status, tumour size, nodal status, differentiation grade, adjuvant treatment). The Cox proportional hazards model was applied for both univariate and multivariate analyses using the associated likelihood ratio test to test for differences. RFS and OS probabilities were calculated by the actuarial method of Kaplan and Meier (1958). For all tests, a two-sided *P*-value below 0.05 was considered to be statistically significant.

### RESULTS

### MRP expression in primary breast cancer

Expression of MRP was determined by IHC with MRPr1 on cryostat sections of primary breast cancer specimens, and the expression was correlated with specific patient and tumour characteristics, and RFS and OS. The MRPr1 antibody reacted abundantly with the MRP-positive control cell line $GLC_4$/ADR, whereas in the parental cell line $GLC_4$, no staining was observed. The $GLC_4$/ADR cells showed membrane staining as well as cytoplasmic staining, as

**Table 4** Univariate Cox regression analysis of overall survival as a function of MRP expression in subgroups of patients

| Variable | Overall survival | | | | | |
|---|---|---|---|---|---|---|
| | n[a] | | RHR[b] | 95% CL[c] | Failures[d] | |
| | MRP positive | MRP negative | | | MRP positive | MRP negative |
| All patients | 87 | 172 | 1.2 | 0.8–1.8 | 33 | 60 |
| Menopausal status | | | | | | |
| Premenopausal | 28 | 62 | 1.2 | 0.5–2.7 | 9 | 19 |
| Post-menopausal | 59 | 110 | 1.1 | 0.7–1.9 | 24 | 41 |
| Tumour size | | | | | | |
| pT1 | 23 | 53 | 2.3 | 0.9–6.0 | 8 | 9 |
| pT2 | 51 | 97 | 1.1 | 0.7–1.9 | 21 | 40 |
| pT3+4 | 12 | 21 | 0.7 | 0.2–2.1 | 4 | 10 |
| Nodal status[e] | | | | | | |
| N0 | 34 | 67 | 1.1 | 0.4–2.6 | 7 | 14 |
| N+ | 53 | 105 | 1.2 | 0.8–2.0 | 26 | 46 |
| Adjuvant treatment | | | | | | |
| *n*+: None | 34 | 63 | 1.0 | 0.5–1.7 | 18 | 36 |
| *n*+: CMF[f] | 7 | 25 | 3.7 | 0.8–17.1 | 3 | 4 |
| *n*+: Hormonal | 11 | 13 | 1.1 | 0.3–4.2 | 5 | 4 |
| ER | | | | | | |
| Negative | 27 | 35 | 1.0 | 0.5–2.2 | 11 | 15 |
| Positive | 60 | 134 | 1.2 | 0.7–1.9 | 22 | 45 |
| PgR | | | | | | |
| Negative | 35 | 52 | 1.2 | 0.6–2.3 | 15 | 20 |
| Positive | 52 | 116 | 1.1 | 0.6–1.9 | 18 | 39 |

[a]*n*, Number of patients. Note that information on all variables was not always available. [b]RHR, relative hazard rate. [c]95% CL, 95% confidence limits. [d]Failures, the number of deaths. [e]*n*+, $n > 0$. [f]CMF: cyclophosphamide, methotrexate and 5-fluorouracil.

© Cancer Research Campaign 1997

**Figure 1** Actuarial relapse-free survival as a function of MRP status in node-negative patients (**A**) and in patients with tumours ≤ 2 cm (**B**). ▬, MRP-positive patients; —, MRP-negative patients. The number of patients at risk is indicated documented previously (Nooter et al, 1995; 1996*a*). The MRP staining among the primary breast carcinomas varied between negative (–) and strong cytoplasmic and membraneous staining of the tumour cells (+++). The specificity of the staining was shown by using an isotype-matched, irrelevant rat MAb (IgG2a subclass) that was always negative. The cytoplasmic and membrane staining of MRP in the tumour cells is consistent with the idea that MRP functions as a membrane-bound drug extrusion pump and is involved in cytoplasmic drug sequestration. Based on staining intensity and cellular localization of the staining, the breast cancer specimens were qualitatively divided into four groups (IHC score: –, +, ++, +++). Expression of MRP (IHC score: +, ++ and +++) was observed in 87 (34%) of the 259 tumour samples studied, while 172 (66%) of the samples had no detectable MRP staining and were scored as negative (–). Forty-nine (19%) of the 259 samples showed weak cytoplasmic staining (+), 35 (14%) had a clear cytoplasmic staining (++), and in only three (1%) a strong cytoplasmic as well as membraneous staining (+++) of tumour cells was observed. The intensity of the staining in the highest MRP staining group (IHC score: +++) equals more or less the intensity observed in the MRP-positive, drug-resistant $GLC_4$/ADR cells. As the intensity of the MRP staining increased, the percentage of stained tumour cells increased also. Tumours with weak cytoplasmic staining (IHC score: +) had mostly between 30–50% of the tumour cells stained, while for the stronger stained tumours (IHC score: ++, +++) this figure was, in general, more than 50%.

### MRP expression in relation to patient and tumour characteristics

In order to assess whether MRP expression at diagnosis was related to patient and tumour characteristics, the patients were stratified into two groups, MRP positive and MRP negative. The MRP-negative group completely lacked MRP expression (IHC score: –), whereas the MRP-positive group comprised all patients with MRP staining (IHC score: + to +++). In Table 1 the percentage of MRP-positive tumours is listed in relation to patient (age and menopausal status), tumour (size and grade of differentiation of the tumour, ER and PgR status and lymph node involvement) and treatment characteristics (adjuyant treatment). No significant differences in MRP staining were detected according to patient's age, menopausal status, tumour size, nodal status, differentiation grade, ER and PgR. In addition, the distribution of MRP expression in tumours of patients who did or did not receive adjuvant therapy was similar.

### MRP expression in relation to relapse-free and overall survival

To evaluate the prognostic significance of MRP expression at diagnosis, MRP expression was analysed in relation to RFS and OS. In a Cox univariate analysis for RFS and OS in all patients, no significant relationship between MRP and RFS and OS was observed (Table 2). Similarly, age, menopausal status, ER and PgR were not significantly related to RFS and OS in univariate analysis. On the other hand, the size of the primary tumour and the number of lymph nodes involved were significantly associated with a shortened RFS and OS (Table 2). In accordance with Cox univariate analysis, multivariate analysis showed that MRP expression was not significantly associated with prognosis.

Subsequently, we performed an exploratory Cox regression analysis for RFS and OS in subgroups of patients stratified by menopausal status, tumour size, nodal status, adjuvant systemic therapy, and ER and PgR status (Tables 3 and 4). For each subgroup, the number of patients positive and negative for MRP are shown. In Tables 3 and 4, the relative risk for failure expressed as RHR with its 95% CL is given in relation to expression of MRP in the primary tumour (MRP positive vs MRP negative). For MRP-negative tumours, the RHR = 1 by definition. We have not given the *P*-values as the power of the Cox regression analysis is low when only a limited number of failures and small groups of patients are available. In three subgroups, MRP expression was associated with increased risk for failure. In patients with small tumours (T1), in node-negative patients and in node-positive patients who received adjuvant systemic chemotherapy with CMF, the RHR for relapse was increased in the presence of MRP (Table 3: RHR 2.8, 2.1 and 2.8 respectively). The relationship of MRP to RFS in the subgroup of patients with small tumours (T1) and in node-negative patients is shown in Figure 1. The numbers of patients at risk are indicated in the figures. In analysis for OS, expression of MRP was also associated with increased risk for failure in patients with small tumours (T1) and in node-positive patients who received adjuvant systemic chemotherapy with CMF but not in node-negative patients (Table 4: RHR 2.3, 3.7 and 1.1 respectively).

© Cancer Research Campaign 1997

### DISCUSSION

A large number of cell biological parameters (reviewed in Klijn et al, 1993), including oncogenes, tumour-suppressor genes, growth factor and hormone receptors, and secretory proteins, have been found to influence tumour behaviour with respect to metastatic pattern, cellular differentiation, growth rate and the development of therapy resistance. In the present study, we determined the expression of the drug-resistance MRP gene in a series of more than 250 primary breast cancer specimens, and we evaluated its expression in relation to patient and tumour characteristics, and RFS and OS. By IHC using the high-affinity MAb MRPr1 (Flens et al, 1994), expression of MRP protein was found in about 30% of primary breast carcinomas. The majority of these samples had weak to moderate MRP expression levels, and only 1% of the primary breast cancer specimens had strong MRP staining in the cytoplasm and on the cell membrane. These results are in agreement with preliminary data from our own group (Nooter et al, 1995) and those of others (Filipits et al, 1996). In a previous study (Nooter et al, 1995), we found expression of *MRP* mRNA, as determined by RNAase protection assay, in approximately 80% of breast cancer specimens. In the same study, MRP protein was found to be expressed in 2 of 11 breast cancer specimens only. In a recent paper by the group of Pirker (Filipits et al, 1996), all primary breast cancer specimens expressed *MRP* mRNA as determined by reverse transcriptase–polymerase chain reaction. By IHC with two MRP-specific MAbs (QCRL-1 and QCRL-3) developed by the group of Cole and Deeley (Hipfner et al, 1994), strong staining was observed in 24% and weak staining in the remaining 76% of the breast cancer specimens (Filipits et al, 1996). From these studies it might be concluded that *MRP* mRNA is expressed at a very low level in most breast cancer specimens and that a smaller part (20–40%) of the specimens have elevated levels of MRP. The ubiquitous, low-level expression of *MRP* mRNA in breast cancer is in concordance with the MRP expression in the normal, unaffected mammary gland (Flens et al, 1996). MRP was detected at the protein level in different types of normal epithelial cells from the bronchus, digestive tract and adrenal cortex (Flens et al, 1996), suggesting that MRP may have an excretory function in protecting the organism against xenobiotics. In the mammary gland, the lobules were negative for MRP, while in the lactiferous ducts some weak, focal expression could be detected.

In the current study, MRP expression at diagnosis was not related to patient's age and menopausal status, tumour size and differentiation grade, ER and PgR level or lymph node involvement. However, in the study by Filipits et al (1996), strong MRP staining was more frequent in T3 and T4 tumours than in T1 and T2 tumours but was also independent of age, menopausal status, histology, histological grade, ER and PgR, and lymph node involvement. We have shown here that, in Cox univariate analysis of all patients, only primary tumour size and the number of lymph nodes involved were significantly associated with a rapid rate of relapse and shorter OS, which is a general finding that has been reported previously by others (reviewed in Harris et al, 1992). Age, menopausal status, ER and PgR status, and MRP expression were not significantly related with the length of RFS and OS. In Cox univariate analysis for RFS in subgroups of patients stratified by menopausal status, tumour size, nodal status, adjuvant systemic therapy, and ER and PgR status, MRP expression was associated with increased risk for failure in patients with small tumours (T1), in node-negative patients and in node-positive patients who received adjuvant systemic chemotherapy with cyclophosphamide, methotrexate and 5-fluorouracil. The rate of relapse was dramatically increased (2.1- to 2.8-fold) in the presence of MRP. In analysis for OS, expression of MRP was also associated with a 2.3-fold increased death rate in patients with small tumours (T1) and a 3.7-fold increased death rate in node-positive patients who received adjuvant systemic chemotherapy with CMF but not in node-negative patients. Apparently, in patients with small tumours and in node-negative patients, MRP expression might be associated with shorter RFS and OS. As these patients had not received any systemic therapy, our data suggest that MRP expression in primary breast cancer might be related to a more aggressive tumour cell behaviour. For the classical 170-kDa Pgp/*MDR1* gene product, such a correlation has also been documented in a variety of different cancers, among which are colon (Weinstein et al, 1991), breast (Linn et al, 1995) and renal cell carcinomas (Tobe et al, 1995). For these cancers, evidence was provided that Pgp expression and tumour invasiveness may be linked. However, the apparent association between Pgp expression and a more malignant phenotype is not a universal phenomenon. In childhood rhabdomyosarcoma, Pgp expression at diagnosis, in fact, appeared to be associated with better RFS and OS (Kuttesch et al, 1996). Nevertheless, together these studies suggest that Pgp might indeed influence tumour cell behaviour.

Although normal tissue distribution and expression of MRP in solid tumours and leukaemias has been documented, so far only limited data are available on the clinical relevance of MRP in human malignancies. Some recent studies (Bordow et al, 1994; Kuss et al, 1994; Ota et al, 1995) suggest, based on historical data, a correlation between clinical response to chemotherapy and level of MRP expression. One study (Kuss et al, 1994) reported the absence of MRP expression as a result of chromosomal aberrations in a subgroup of drug-sensitive (daunorubicin and ara-C) AML (M4), with inversion of chromosome 16. Another study (Bordow et al, 1994) suggested the complementary correlation of increased *MRP* expression in aggressive, notorious drug-resistant neuroblastomas with N-*myc* oncogene amplification. Expression of the *MRP* gene was correlated with amplification and overexpression of the N-*myc* oncogene, especially in advanced-stage tumours that tend to be particularly aggressive and unresponsive to chemotherapy. In squamous cell carcinoma of the lung, the prognoses of patients with MRP expression were significantly worse than those of patients without MRP expression (Ota et al, 1995). In the present study, MRP expression in adjuvant CMF-treated, node-positive patients was associated with duration of RFS and OS, suggesting that MRP expression might encode drug resistance in vivo against adjuvant systemic chemotherapy. In this respect, it is of note that as yet none of the drugs in the CMF regimen has been shown to be a substrate for MRP. In particular, in analysis for OS, the absence of MRP expression was associated with a prolonged survival. Of these node-positive CMF-treated patients, 92% were still alive after 5 years compared with 57% for the patients with a positive MRP score. Although very suggestive, these data are based on small patient numbers and should therefore be considered carefully; they should rather be used as indication for future studies. In conclusion, our results show that MRP is frequently overexpressed in primary breast cancer and suggest that MRP expression might be of prognostic significance in the subgroups of patients with a more favourable prognosis, i.e. patients with small tumours and node-negative patients, as well as in the setting of adjuvant systemic chemotherapy. Further studies with larger

© Cancer Research Campaign 1997

patient populations should confirm whether in primary breast cancer MRP is related to altered cell biological behaviour, including a more aggressive phenotype, and resistance to adjuvant systemic chemotherapy.

### ACKNOWLEDGEMENTS

We thank Dr EGE de Vries (University of Groningen, Groningen, The Netherlands) for providing cell lines and Mrs CJC Claassen and ME Meijer-van Gelder for technical assistance. This work was supported by the Dutch Cancer Society (Grants DDHK95-1051 and DDHK92-04).

### REFERENCES

Bordow SB, Haber M, Madafiglio J, Cheung B, Marshall GM and Norris MD (1994) Expression of the multidrug resistance-associated protein (MRP) gene correlates with amplification and overexpression of the N-myc oncogene in childhood neuroblastoma. *Cancer Res* **54**: 5036–5040

Breuninger LM, Paul S, Gaughan K, Miki T, Chan A, Aaronson SA and Kruh GD (1995) Expression of multidrug resistance-associated protein in NIH/3T3 cells confers multidrug resistance associated with increased drug efflux and altered intracellular drug distribution. *Cancer Res* **55**: 5342–5347

Burger H, Nooter K, Zaman GJR, Sonneveld P, Van Wingerden K, Oostrum RG and Stoter G (1994*a*) Expression of the multidrug resistance-associated protein (MRP) in acute and chronic leukemias. *Leukemia* **8**: 990–997

Burger H, Nooter K, Sonneveld P, Van Wingerden K, Zaman GJR and Stoter G (1994*b*) High expression of the multidrug resistance-associated protein (MRP) in chronic and prolymphocytic leukemia. *Br J Haematol* **88**: 348–356

Childs S and Ling V (1994) The MDR superfamily of genes and its biological implications. In *Important Advances in Oncology 1994*, DeVita VT, Hellman S and Rosenberg SA. (eds), pp. 21–36. Lippincott: Philadelphia

Clynes M (1993) *Multiple Drug Resistance in Cancer. Cellular, Molecular and Clinical Approaches*. Kluwer: Dordrecht

Cole SPC, Bhardwaj G, Gerlach JH, Mackie JE, Grant CE, Almquist KC, Stewart AJ, Kurz EU, Duncan AMV and Deeley RG (1992) Overexpression of a transporter gene in a multidrug-resistant human lung cancer cell line. *Science* **258**: 1650–1654

Cole SPC, Sparks KE, Fraser K, Loe DW, Grant CE, Wilson GM and Deeley RG (1994) Pharmacological characterization of multidrug resistant MRP-transfected human tumour cells. *Cancer Res* **54**: 5902–5910

Doyle AL (1993) Mechanisms of drug resistance in human lung cancer. *Semin Oncol* **20**: 326–337

Endo K, Maehara Y, Ichiyoshi Y, Kusumoto T, Sakaguchi Y, Ohno S and Sugimachi K (1996) Multidrug resistance-associated protein expression in clinical gastric carcinoma. *Cancer* **77**: 1681–1687

EORTC Breast Cancer Cooperative Group (1980) Revision of the standards for the assessment of hormone receptors in human breast cancer. *Eur J Cancer* **16**: 1513–1515

Filipits M, Suchomel RW, Dekan G, Haider K, Valdimarsson G, Depisch D and Pirker R (1996) MRP and MDR1 gene expression in primary breast carcinomas. *Clin Cancer Res* **2**: 1231–1237

Flens MJ, Izquierdo MA, Scheffer GL, Fritz JM, Meijer CJLM, Scheper RJ and Zaman GJR (1994) Immunochemical detection of multidrug resistance-associated protein MRP in human multidrug-resistant tumor cells by monoclonal antibodies. *Cancer Res* **54**: 4557–4563

Flens MJ, Zaman GJR, Van Der Valk P, Izquierdo MA, Schroeijers AB, Scheffer GL, Van Der Groep P, De Haas M, Meijer CJLM and Scheper RJ (1996) Tissue distribution of the multidrug resistance protein. *Am J Pathol* **148**: 1237–1247

Foekens JA, Portengen H, Van Putten WLJ, Peters HA, Krijnen HLJM, Alexieva-Figusch J and Klijn JGM (1989) Prognostic value of estrogen and progesterone receptors measured by enzyme immunoassays in human breast tumor cytosols. *Cancer Res* **54**: 5823–5828

Goldstein LJ and Ozols RF (1994) *Anticancer Drug Resistance: Advances in Molecular and Clinical Research*. Kluwer: Boston

Grant CE, Valdimarsson G, Hipfner DR, Almquist KC, Cole SPC and Deeley RG (1994) Overexpression of multidrug resistance-associated protein (MRP) increases resistance to natural product drugs. *Cancer Res* **54**: 357–361

Harris JR, Lippman ME, Veronesi U and Willett W (1992) Breast cancer. *N Engl J Med* **327**: 319–328, 390–398, 473–480

Henderson IC, Harris JR, Kinne DW and Hellman S (1989) Cancer of the breast. In *Cancer, Principles and Practice of Oncology*, Devita VT, Hellman S and Rosenberg SA. (eds), pp. 1197–1268. Lippincott: Philadelphia

Hipfner DR, Gauldie SD, Deeley RG and Cole SPC (1994) Detection of the *Mr* 190,000 multidrug resistance protein, MRP, with monoclonal antibodies. *Cancer Res* **54**: 5788–5792

Kaplan EL and Meier P (1958) Non-parametric estimation from incomplete observations. *J Am Stat Assoc* **53**: 457–481

Kavallaris M, Leary JA, Barrett JA and Friedlander ML (1996) MDR1 and multidrug resistance-associated protein (MRP) gene expression in epithelial ovarian tumors. *Cancer Lett* **102**: 7–16

Klijn JGM, Berns EMJJ, Bontebal M and Foekens J (1993) Cell biological factors associated with the response of breast cancer to systemic treatment. *Cancer Treat Rev* **19**: 45–63

Krishnamachary N and Center MS (1993) The *MRP* gene associated with a non-P-glycoprotein multidrug resistance encodes a 190-kDa membrane bound glycoprotein. *Cancer Res* **53**: 3658–3661

Kruh GD, Chan A, Myers K, Gaughan K, Miki T and Aaronson SA (1994) Expression complementary DNA library transfer establishes mrp as a multidrug resistance gene. *Cancer Res* **54**: 1649–1652

Kuss BJ, Deeley RG, Cole SPC, Willman CL, Kopecky KJ, Wolman SR, Eyre HJ, Lane SA, Nancarrow JK, Whitmore SA and Callen DF (1994) Deletion of gene for multidrug resistance in acute myeloid leukemia with inversion in chromosome 16: prognostic implications. *Lancet* **343**: 1531–1534

Kuttesch JF, Parham DM, Luo X, Meyer WH, Bowman L, Shapiro DN, Pappo AS, Crist WM, Beck WT and Houghton PJ (1996) P-glycoprotein expression at diagnosis may not be a primary mechanism of therapeutic failure in childhood rhabdomyosarcoma. *J Clin Oncol* **14**: 886–900

Lai SL, Goldstein LJ, Gottesman MM, Pastan I, Tsai CM, Johnson BE, Mulshine JL, Ihde DC, Kayser K and Gazdar AF (1989) MDR1 gene expression in lung cancer. *J Natl Cancer Inst* **81**: 1144–1150

Linn SC, Giaccone G, Van Diest PJ, Blokhuis WMD, Van Der Valk P, Van Kalken CK, Kuiper CM, Pinedo HM and Baak JPA (1995) Prognostic relevance of P-glycoprotein expression in breast cancer. *Ann Oncol* **6**: 679–685

McGuire WL and Clark GM (1992) Prognostic factors and treatment decisions in axillary-node-negative breast cancer. *N Engl J Med* **326**: 1756–1761

Merkel DE, Fuqua SAW, Tandon AK, Hill SM, Buzdar AV and McGuire WL (1989) Electrophoretic analysis of 248 clinical breast cancer specimens for P-glycoprotein overexpression or amplification. *J Clin Oncol* **7**: 1129–1136

Nooter K and Sonneveld P (1994) Clinical relevance of P-glycoprotein expression in hematological malignancies. *Leukemia Res* **18**: 233–243

Nooter K, Westerman A, Flens MJ, Zaman GJR, Scheper RJ, Van Wingerden KE, Burger H, Oostrum R, Boersma T, Sonneveld P, Gratama JW, Kok T, Eggermont AMM, Bosman FT and Stoter G (1995) Expression of the multidrug resistance-associated protein (MRP) gene in human cancers. *Clin Cancer Res* **1**: 1301–1310

Nooter K, Bosman FT, Burger H, Van Wingerden KE, Flens MJ, Scheper RJ, Oostrum RG, Boersma AWM, Van Der Gaast A and Stoter G (1996*a*). Expression of the multidrug resistance-associated protein (MRP) gene in primary non-small-cell lung camcer. *Ann Oncol* **7**: 75–81

Nooter K, Burger H and Stoter G (1996*b*) Multidrug resistance-associated protein (MRP) in haematological malignancies. *Leuk Lymphoma* **20**: 381–387

Ota E, Abe Y, Oshika Y, Ozeki Y, Iwasaki M, Inoue H, Yamazaki H, Ueyama Y, Takagi K, Ogata T, Tamaoki N and Nakamura M (1995) Expression of the multidrug resistance-associated protein (MRP) gene in non-small-cell lung cancer. *Br J Cancer* **72**: 550–554

Paul S, Breuninger LM, Tew KD, Shen H and Kruh GD (1996) ATP-dependent uptake of natural product cytotoxic drugs by membrane vesicles establishes MRP as a broad specificity transporter. *Proc Natl Acad Sci USA* **93**: 6929–6934

Schneider E, Cowan KH, Bader H, Toomey S, Schwartz GN, Karp JE, Burke PJ and Kaufmann SH (1995) Increased expression of the multidrug resistance-associated protein gene in relapsed acute leukemia. *Blood* **85**: 186–193

Thomas GA, Barrand MA, Stewart S, Rabbitts PH, Williams ED and Twentyman PR (1994) Expression of the multidrug resistance-associated protein (MRP) gene in human lung tumours and normal tissues as determined by *in situ* hybridisation. *Eur J Cancer* **30A**: 1705–1709

Tobe SW, Noble-Topham SE, Andrulis IL, Warren R, Hartwick J, Skorecki KL and Warner E (1995) Expression of the multiple drug resistance gene in human renal cell carcinoma depends on tumour histology, grade, and staging. *Clin Cancer Res* **1**: 1611–1615

Weinstein RS, Jakate SM, Dominguez JM, Lebovitz MD, Koukoulis GK, Kuszak JR, Klusens LF, Grogan TM, Saclarides TJ, Roninson IB and Coon JS (1991)

 © Cancer Research Campaign 1997

Relationship of the expression of the multidrug resistance gene product (P-glycoprotein) in human colon carcinoma to local tumor aggressiveness and lymph node metastasis. *Cancer Res* **51**: 2720–2726
Zaman GJR, Versantvoort CHM, Smit JJM, Eijdems EWHM, De Haas M, Smith AJ, Broxterman HJ, Mulder NH, De Vries EGE, Baas F and Borst P (1993) Analysis of the expression of MRP, the gene for a new putative transmembrane drug transporter, in human multidrug resistant lung cancer cell lines. *Cancer Res* **53**: 1747–1750
Zaman GJR, Flens MJ, Van Leusden MR, De Haas M, Mulder HS, Lankelma J, Pinedo HM, Scheper RJ, Baas F, Broxterman HJ and Borst P (1994) The human multidrug resistance-associated protein MRP is a plasma membrane drug-efflux pump. *Proc Natl Acad Sci USA* **91**: 8822–8826

© Cancer Research Campaign 1997